# *Dudleya brittonii* extract promotes survival rate and M2-like metabolic change in porcine 3D4/31 alveolar macrophages

**DOI:** 10.5713/ajas.19.0251

**Published:** 2019-05-27

**Authors:** Hyungkuen Kim, Eek Hyung Jeon, Byung-Chul Park, Sung-Jo Kim

**Affiliations:** 1 Division of Cosmetics and Biotechnology, College of Life and Health Sciences, Hoseo University, Baebang, Asan 31499, Korea; 2 Department of Biotechnology and Bioinformatics, College of Science and Technology, Korea University, Sejong 30019, Korea; 3 Graduate School of International Agricultural Technology and Institute of Green-Bio Science and Technology, Seoul National University, Pyeongchang 25354, Korea

**Keywords:** Porcine, Alveolar Macrophage, *Dudleya brittonii*, Reactive Oxygen Species, Fatty Acid, Immunity

## Abstract

**Objective:**

Although alveolar macrophages play a key role in the respiratory immunity of livestock, studies on the mechanism of differentiation and survival of alveolar macrophages are lacking. Therefore, we undertook to investigate changes in the lipid metabolism and survival rate, using 3D4/31 macrophages and *Dudleya brittonii* which has been used as a traditional asthma treatment.

**Methods:**

3D4/31 macrophages were used as the *in vitro* porcine alveolar macrophages model. The cells were activated by exposure to phorbol 12-myristate 13-acetate (PMA). *Dudleya brittonii* extraction was performed with distilled water. For evaluating the cell survival rate, we performed the water-soluble tetrazolium salt cell viability assay and growth curve analysis. To confirm cell death, cell cycle and intracellular reactive oxygen species (ROS) levels were measured using flow cytometric analysis by applying fluorescence dye dichlorofluorescein diacetate and propidium iodide. Furthermore, we also evaluated cellular lipid accumulation with oil red O staining, and fatty acid synthesis related genes expression levels using quantitative polymerase chain reaction (qPCR) with SYBR green dye. Glycolysis, fatty acid oxidation, and tricarboxylic acid (TCA) cycle related gene expression levels were measured using qPCR after exposure to *Dudleya brittonii* extract (DB) for 12 h.

**Results:**

The ROS production and cell death were induced by PMA treatment, and exposure to DB reduced the PMA induced downregulation of cell survival. The PMA and DB treatments upregulated the lipid accumulation, with corresponding increase in the acetyl-CoA carboxylase alpha, fatty acid synthase mRNA expressions. DB-PMA co-treatment reduced the glycolysis genes expression, but increased the expressions of fatty acid oxidation and TCA cycle genes.

**Conclusion:**

This study provides new insights and directions for further research relating to the immunity of porcine respiratory system, by employing a model based on alveolar macrophages and natural materials.

## INTRODUCTION

Worldwide rapid climate changes (heat wave, fine dust, etc.) and high rates of dense breeding have negatively affected livestock immunity [1–3]. This trend has been exacerbated by the prohibition of growth-promoting hormones and antibiotics in livestock breeding [4]. Compared to cancer or dementia, respiratory infectious diseases that spread easily are of graver concern for farmhouses. This has resulted in increased importance focused on livestock health and respiratory immunity [5].

In general, blood circulating monocytes (CMs) are derived from bone marrow, while alveolar macrophages (AMs) are specific tissue macrophages which form a few days after birth; briefly, fetal monocytes differentiate into AMs and settle in the alveolar tissue [6]. In addition, AMs encompass a self-renewal ability, which maintains the characteristics of existing AMs and embraces alveolar characteristics [7]. The CMs from bone marrow helps restore the number of AMs when the population decreases due to severe infection or radiotherapy, etc. [8]. The specific characteristics of AMs implies that CM-based studies are not applicable to AMs. Hence, using AMs such as 3D4/31, are more appropriate to study pulmonary health and immunity.

Previously, classical M1 macrophages were closely associated with the glycolysis pathway, which is a key regulator of macrophage activation. However, recent studies on M2-like macrophages such as AMs report that lipid metabolism plays an important role in macrophage differentiation and activation of the M2 form. Two basic lipid metabolisms (fatty acid synthesis [FAS] and fatty acid oxidation [FAO]) are known to work centrally [9]. The FAS pathway can be summarized as follows: carboxylation of acetyl-CoA to malonyl-CoA by acetyl-CoA carboxylase alpha (ACACA), and the production of palmitic acid (most basic fatty acids found in mammals) from malonyl-CoA or acetyl-CoA by fatty acid synthase (FASN) [10]. Using these or externally absorbed lipids, adenosine triphosphate (ATP) is produced through the FAO and tricarboxylic acid (TCA) cycle. The FAO pathway functions by converting fatty-acyl-CoA to acyl-CoA by the action of carnitine palmitoyltransferase (CPT), subsequent to beta-oxidation in the mitochondrial matrix by several enzymes include acetyl-CoA acyltransferase (ACAA), and finally production of acetyl-CoA by β-oxidation, and involvement in the TCA-cycle for generating ATP [11].

Macrophage also have signaling to regulates excessive lipid accumulation. Enhanced steroid receptor coactivator-3 (SRC3) signaling promotes the metastasis of lung cancer and myeloma cells, which are known to be fatal in patients [12,13]. However, SRC3 plays an essential role in lipid metabolism and macrophage survival, by initiating FAO and preventing excessive fatty acid accumulation by reacting sensitively to fatty acid concentration [14]. Moreover, SRC3 signaling is activated by infection, thereby increasing the expression of antioxidant enzymes (catalase) and anti-apoptotic B-cell lymphoma 2 to reduce reactive oxygen species (ROS) generation and cell death, and improve bacterial clearance [15]. However, this insight into the AMs has not yet been achieved.

Plants are one of the most remarkable materials for enhancing livestock immunity and replacing antibiotics with low toxicity and various functionalities [16]. Since the research on the medical efficacy of plants has been continuously carried out and most of the plants have been studied, there is a growing demand for new plant species that have not been studied previously. The succulent plant, represented by aloe, is inhabited in unique environments such as desert, tropical regions, and coasts with high salinity, and has undergone relatively less research than other plant species [17]. Nevertheless, a small number of succulent plants, such as aloe and prickly pear, have been successfully used for commercial purposes because of their proven efficacy in immunity [18,19]. For this study, we selected *Dudleya brittonii* (*D. brittonii*) from several succulent plant species by collecting various information about respiratory immunity. *D. brittonii*, also called giant chalk dudleya, is a succulent plant mainly found in California and Mexico, and is widely used for ornamental purposes [20]. Due to a dearth of biological studies on *D. brittonii*, the effect on cells or animals is yet to be elucidated. Interestingly, according to the University of San Diego, Kumeyaay, a native American in California, used *D. brittonii* for treating asthma, an inflammatory airway obstruction disease closely associated with macrophages. Therefore, in this study, we attempt to verify the survival and metabolic efficacy of *D. brittonii* extract (DB) in AMs.

## MATERIALS AND METHODS

### Preparation of *Dudleya brittonii* extract

Thirty grams of *D. brittonii* leaves were mixed with 300 mL of distilled water (DW), and extraction was performed at 110°C and 39.23 kPa for 15 min in an autoclave (Daihan Scientific, Wonju, Korea). The aqueous phase was collected and filtered with a 0.2 μm syringe filter (Minisart, Sartorius, Goettingen, Germany).

### Cell culture

3D4/31 macrophages (ATCC-CRL-2844; ATCC, Manassas, VA, USA) were cultured in complete Roswell Park Memorial Institute (RPMI) 1640 medium (supplemented with 10% [v/v] fetal bovine serum and 1% [v/v] penicillin streptomycin) in a CO_2_ incubator (95% air and 5% CO_2_, 37°C).

### Cell survival rate analysis

To measure the survival rate, water-soluble tetrazolium salt (WST) -1 cell viability assay and growth curve analysis were performed, as described previously [21]. Briefly, 3×10^3^ 3D4/31 macrophages were seeded in 96-well cell culture plates (SPL Life Sciences, Pocheon, Korea); after 24 h, cells were treated with DB (900, 9, 0.09 ng/mL) and/or 2 nM phorbol 12-myristate 13-acetate (PMA; Sigma-Aldrich, St. Louis, MO, USA), in triplicates. To determine cell viability, WST-1 reagent (EZ Cytox Cell Viability Assay Kit; DoGenBio, Seoul, Korea) was added to each well, and absorbance was measured at 450 nm using a microplate reader (Sunrise, Tecan, Männedorf, Switzerland). Each absorbance was compared to the vehicle control. For growth curve analysis, 5×10^4^ cells were seeded in 60-mm cell culture dishes (SPL Life Sciences, Korea) and incubated for 24 h. Cells were then exposed to DB (9 ng/mL), with/without 2 nM PMA, and fresh media was replenished every 24 h. Cell numbers were assessed every 24 h using a hemocytometer and 0.4% (w/v) trypan blue (Thermo Fisher Scientific, Waltham, MA, USA) staining. Microscopic analysis was performed using the CKX41 microscope (Olympus Optical Co., Center Valley, PA, USA) and AxioCam HRc (Carl Zeiss, Oberkochen, Germany). Images were processed with the Axio vision Rel. 4.7 (Carl Zeiss) and Photoshop CC 2018 program (Adobe Systems, San Jose, CA, USA).

### Oxidative stress analysis

Intracellular ROS levels of 3D4/31 macrophages were measured using the ROS indicator dichlorofluorescein diacetate (DCFDA, Sigma-Aldrich, USA), as described before [21]. Briefly, 5×10^5^ cells were seeded in a 60-mm cell culture dish. After 12 h, cells were exposed to DB (900, 9, 0.09 ng/mL) with/without 2 nM PMA, using DW as vehicle, followed by 12 h incubation. DCFDA (final concentration of 10 μM) was administered for 30 min, after which the cells were harvested and washed with phosphate buffered saline (PBS; pH 7.4 ). DCF-median fluorescence intensity was detected by the Guava EasyCyte (Millipore, Temecula, CA, USA) and FlowJo (TreeStar, Ashland, OR, USA), and relative ROS levels were measured as compared to vehicle.

### Cell cycle analysis

Propidium iodide (PI; Sigma-Aldrich, USA) was used for cell cycle analysis, as described before [22]. Briefly, 5×10^5^ 3D4/31 macrophages were seeded in a 60-mm cell culture dish and incubated for 12 h. The cells were treated with DB (9 ng/mL) with/without 2 nM PMA for 12 h. Treated cells were then fixed and permeabilized using ice-cold ethanol at −20°C, washed, and resuspended in PBS, followed by addition of RNase A (final concentration at 250 μg/mL). After 30 min incubation room temperature (22°C), cells were stained with PI solution (final concentration at 500 μg/mL) for 15 min in the dark at 4°C, PI fluorescence intensity was measured using Guava EasyCyte and FlowJo.

### Lipid staining and concentration measurements

Oil red O was used for lipid staining, as described before, with minor modification [23]. Briefly, 5×10^5^ 3D4/31 macrophages were seeded in a 60-mm cell culture dish. After 12 h, cells were treated with DB (9 ng/mL) and/or 2 nM PMA for 24 h. The cells were washed with PBS and fixed in 3.8% formaldehyde for 15 min, washed with 60% (v/v) isopropanol, air dried, and stained with oil red O (Sigma Aldrich, USA; 0.2 μm filtered, 0.3% [w/v] oil red O in 60% [v/v] isopropanol). Stained cells were then washed with DW and examined for oil red O staining using the DMi8 fluorescence microscope (Leica, Deerfield, IL, USA). Images were processed by applying LAS X (Leica, USA) and Photoshop CC 2018 program.

### Quantitative polymerase chain reaction

Briefly, 5×10^5^ cells 3D4/31 macrophages were seeded in a 60-mm cell culture dish for RNA isolation. After 24 h, cells were exposed to DB (9 ng/mL) with/without 2 nM PMA, and incubated for further 12 h. Total RNA was extracted using TRIzol (Thermo Fisher Scientific, USA), according to the manufacturer’s manual. cDNA synthesis of 3D4/31 macrophages was performed using the MLV-RT cDNA synthesis kit (Wizbiosolutions, Seongnam, Korea) as per the manufacture’s manual. Total 10 ng of cDNA was used for quantitative polymerase chain reaction (qPCR) using the 2X SYBR Green PCR Master Mix (Biofact, Daejeon, Korea), performed according to the manufacturer’s instructions. Specific primer sequences are described in Table 1. The qPCR was performed by using the StepOnePlus Real-Time PCR System (Applied Biosystems, Foster City, CA, USA), and mRNA fold change was analyzed using the 2^−ΔΔCt^ method [24].

### Statistical analysis

All experiments were repeated at least three times and data were obtained from three separate experiments. All data are expressed as the mean±standard deviation (SD). Analysis was performed by GraphPad PRISM 8 (GraphPad Software, San Diego, CA, USA) and Microsoft Excel (Office 365; Microsoft, Redmond, WA, USA). Two-tailed Student’s *t*-test was used to measuring the p-value. Data are considered statistically significant when the p-value is <0.05.

## RESULTS

### *Dudleya brittonii* extract protect 3D4/31 macrophages against phorbol phorbol 12-myristate 13-acetate induced cell death

**Figure 1:**
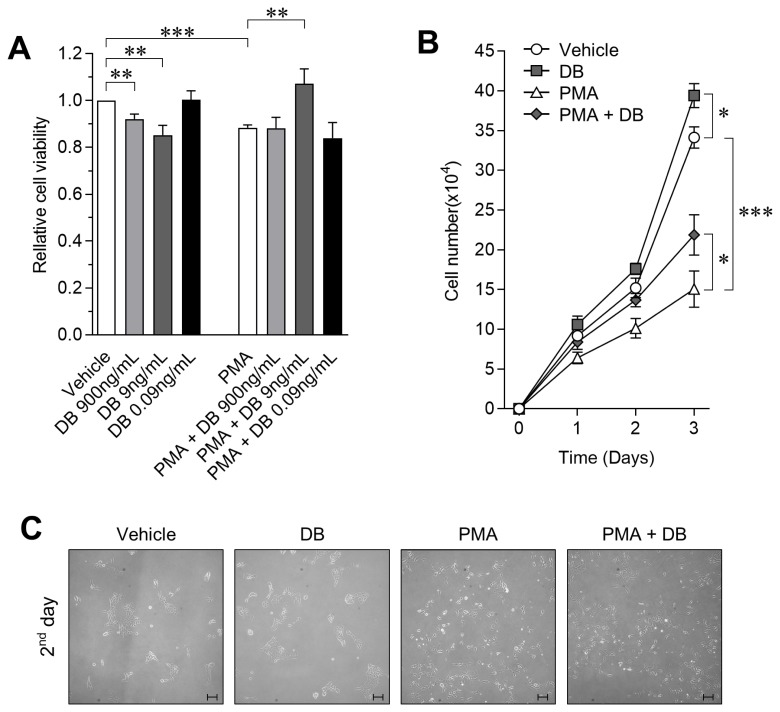
Inhibitory effect of DB on decreased PMA-induced cell survival rate in 3D4/31. (A) Relative cell viability of 3D4/31 macrophages after treatment with DB and/or PMA (2 nM) for 24 h. (B,C) Growth curve analysis of 3D4/31 macrophages; DB (9 ng/mL), and PMA (2 nM) replaced every 24 h. Cell number (B) and phenotype (C) of 3D4/31 macophages (×10, scale bar = 100 μm). DB, *Dudleya brittonii* extract; PMA, phorbol 12-myristate 13-acetate. All data represent the mean±standard deviation (n = 3); * p<0.05, ** p<0.01, *** p<0.001.

To test the protective effect of DB on decreasing cell viability in activated macrophages, we performed the WST-1 assay after exposing cells to DB or PMA for 24 h. As compared to vehicle control, we observed a dose-dependent decrease in viability after exposure to DB, and viability was decreased by 14.77%±4.2% after PMA treatment. However, co-treatment with DB and PMA for 24 h significantly increased the cell viability by up to 11.87%±4.62% as compared to PMA treated cells (Figure 1A; p<0.01). The WST-1 cell viability assay showed a dose-dependent viability-enhancing effect of DB on activated 3D4/31 macrophages, and confirmed the highest activity at 9 ng/mL concentration. Therefore, we performed the growth curve analysis at a final concentration of 9 ng/mL to confirm the effect of DB on the proliferation rate of 3D4/31 macrophages. In this assay, cells were exposed to DB with/without PMA every 24 h. We observed that PMA decreases the cell number by 44.14%±6.66% as compared to vehicle, but co-treatment with DB increases the cell number by 45.13%±16.81% as compared to PMA treated cells (Figure 1B; p<0.05). In addition, the phenotype was observed to confirm for the inhibitory effect of DB on 3D4/31 macrophages activation. No specific phenotypic difference was observed between the PMA-treated and PMA-DB co-treated groups (Figure 1C).

Our results indicate that the survival rate of 3D4/31 macrophages was dramatically decreased when activated by PMA, and DB effectively prevents the PMA-induced inhibition of survival rate. Unlike M1 macrophages (which are mainly involved in phagocytosis), AMs (M2 type) survive longer and are important for wound healing and anti-inflammation [25]. Thus, we confirmed that the survival protection efficacy of DB is associated with respiratory immunity controlled by AMs.

### *Dudleya brittonii* extract exerts anticytotoxic and antioxidant effect in phorbol 12-myristate 13-acetate treated cells

**Figure 2:**
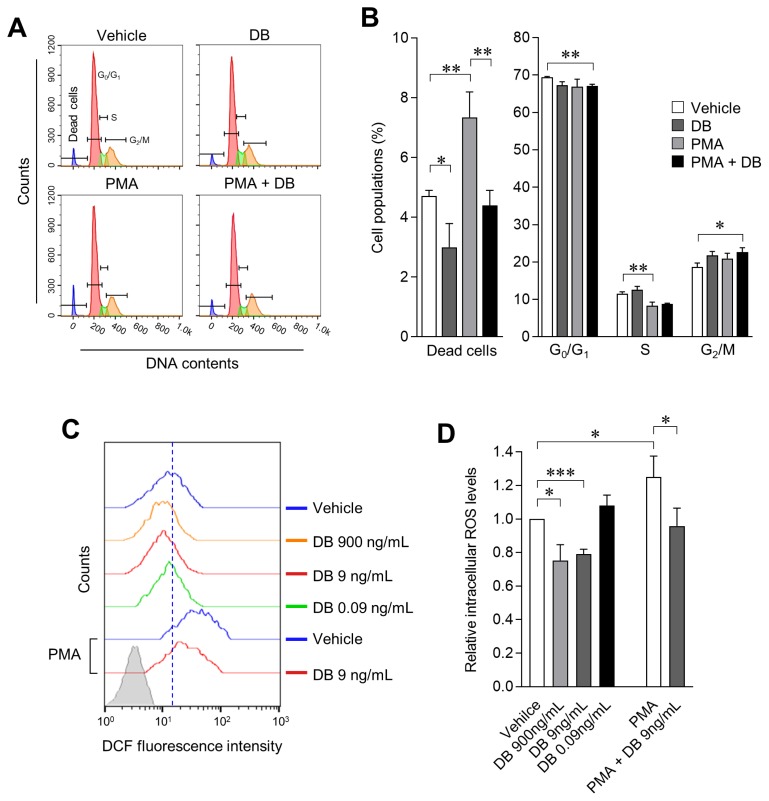
Anticytotoxic and antioxidant effects of DB on PMA treated 3D4/31 macrophages. (A, B) Cell cycle analysis of 3D4/31 macrophages: DB (9 ng/mL) and/or PMA (2 nM) was treated for 24 h. Based on flow cytometric profiles (A), dead cell populations (B) are increased by PMA treatment and reduced after DB exposure. (C, D) Intracellular ROS levels of 3D4/31 macrophages, after exposure to DB or/and PMA (2 nM) for 12 h. Representative flow cytometric profiles (C) and relative intracellular ROS levels (D), was measured via flow cytometry with DCF-DA. DB, *Dudleya brittonii* extract; PMA, phorbol 12-myristate 13-acetate; ROS, reactive oxygen species; DCF-DA, dichlorofluorescein diacetate. All data represent the mean±standard deviation (n = 3); * p<0.05, ** p<0.01, *** p<0.001.

Inhibition of cell survival rate by macrophage activation results from excessive changes in the cell cycle and cell death due to excessive ROS production. We therefore examined the effect of DB on the cell cycle of macrophages treated with/without PMA; cell cycle and cell death were evaluated by PI staining and flow-cytometric analysis. We observed that DB does not induce any significant change in the cell cycle either by applying single treatment or co-treatment with PMA, in 3D4/31 macrophages. However, the dead cells population was increased 2-fold as compared to the vehicle when DB was co-treated with PMA; furthermore, exposure to DB also reduces the number of dead cells as compared to the vehicle only treated cells (Figure 2A, 2B; p<0.05). These results demonstrate that DB reduces the cell death in activated macrophages, and provides evidence of enhanced cell survival effect after DB exposure. We simultaneously confirmed the effects of DB on ROS production (12 h after treatment with DB and/or PMA). DCFDA, a plasma membrane permeable molecule and sensitive indicator of ROS, was applied for the investigation. We observed intracellular reduction of ROS levels (20.93%±2.89%) in 3D4/31 macrophages after treatment with DB, as compared to vehicle treatment and PMA treatment (25.08%±12.41% increase after PMA treatment). Furthermore, DB-PMA co-treatment reduced the ROS level by 23.43%± 8.54% as compared to PMA treatment (Figure 2C, 2D; p< 0.05). Taken together, these results indicate that the PMA induced 3D4/31 macrophage activation induces ROS production, and DB has an antioxidant effect on both non-activated and PMA-activated 3D4/31 macrophages.

Activated M1 macrophage ROS plays an important role in phagocytosis, but excessive ROS production induces inflammation and apoptosis by activating pro-inflammatory signaling such as tumor necrosis factor-α and nuclear factor κ-light-chain-enhancer of activated B cells [26]. However, M2-like AMs do not require excessive ROS production to exert their functions, thus activating the antioxidant system even further to lower the ROS level. Therefore, we believe that the antioxidant effect of DB on 3D4/31 macrophages will have a positive effect on the function and lifespan of M2-type AMs.

### *Dudleya brittonii* extract treatment induces lipid accumulation

**Figure 3:**
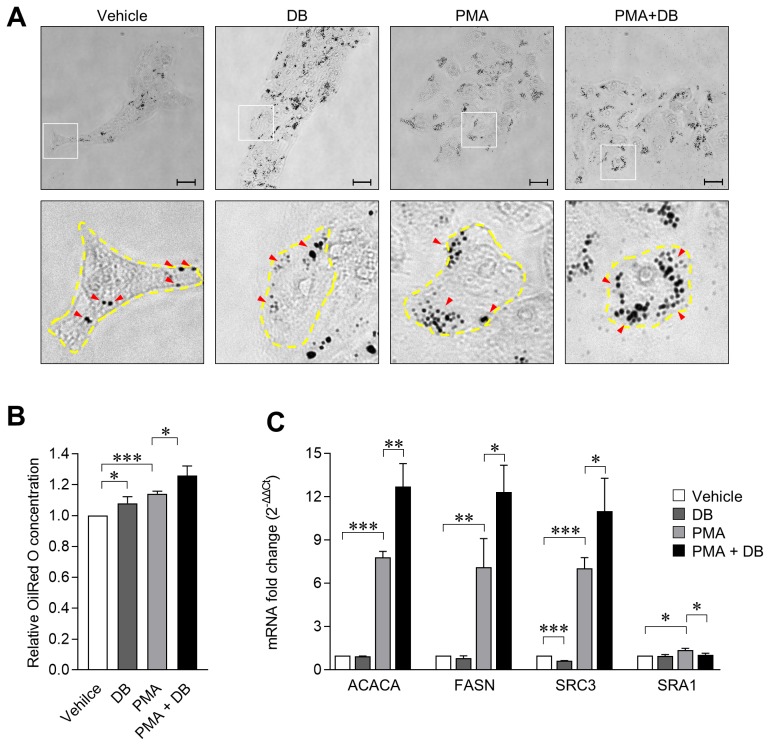
DB upregulates the fatty acid synthesis in 3D4/31 macrophages. (A) Oil red O staining of 3D4/31 macrophages after treatment with DB (9 ng/mL) and/or PMA (2 nM) for 24 h (×20, scale bar = 25 μm). Yellow line: single cell area, red arrow: oil red O stained area. (B) Relative oil red O concentration (O.D 492 nm). Lipid synthesis and accumulation increases after exposure to DB and PMA. (C) qPCR analysis of fatty acid synthesis related genes. DB, *Dudleya brittonii* extract; PMA, phorbol 12-myristate 13-acetate; qPCR, quantitative polymerase chain reaction. All data represent the mean±standard deviation (n = 3); * p<0.05, ** p<0.01, *** p<0.001.

After demonstrating that DB effectively inhibits 3D4/31 macrophage cell death induced by exposure to PMA, we anticipated the possibility that DB may interfere with the activation of macrophages or cause metabolic problems in 3D4/31 macrophages. Since activated M2-like macrophages focus on lipid metabolism, we investigated the effect of DB on lipid metabolism of 3D4/31 macrophages by investigating for lipid production and its associated mechanism. To accomplish this, we measured lipid production by oil red O staining after 24 hours of DB and/or PMA treatment. DB (7.91%± 4.29%) and PMA (14.07%±1.72%) treatments increased the lipid production of 3D4/31 macrophages, and the highest increase (25.92%±6.16%) was confirmed after co-treatment of PMA and DB (Figure 3A, 3B; p<0.05). This confirms that DB has a positive effect on the lipid metabolism of 3D4/31 macrophages.

In addition, we confirmed alterations of FAS pathway genes expression in 3D4/31 macrophages by qPCR, after treatment with DB and/or PMA for 12 h. PMA treatment upregulated the mRNA expression of ACACA and FASN, which are both crucial in the FAS pathway, and expression levels of ACACA and FASN were greater under conditions of co-treatment as compared to PMA single treatment. These results indicate that DB enhances the FAS of PMA-activated 3D4/31 macrophages by controlling the ACACA and FASN expression (Figure 3C; p<0.05). In addition, expression of SRC3 was increased by PMA and DB-PMA co-treatment, suggesting induction of M2-like differentiation of 3D4/31 macrophages, and normal functioning of the sensor mechanism to regulate lipogenesis [27].

Unlike M1 macrophages which mainly utilize glycolysis, M2-like macrophages use the lipid metabolism. However, there are few studies reporting the changes in lipid production of porcine AMs. In this study, we confirm that PMA and DB upregulate the FAS pathway of porcine AMs, and increase lipid accumulation. Therefore, our results indicate that PMA induced activation is accompanied by lipid accumulation of porcine lung macrophages. In addition, since FAS plays a key role in phagocytic differentiation in monocytes [28] (a macrophage precursor cell), it is expected that administration of DB does not adversely affect the anti-bacterial function even if lipid production is upregulated in porcine CMs.

### *Dudleya brittonii* extract treatment results in fatty acid oxidation biased metabolic change in 3D4/31 macrophages

**Figure 4:**
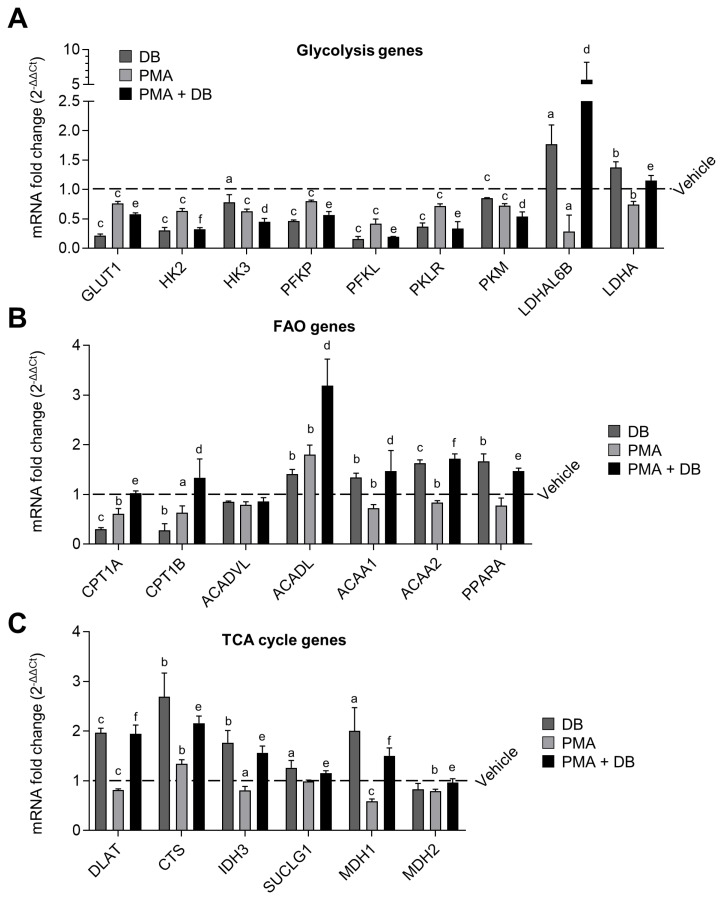
M2-like metabolic reprograming by DB in 3D4/31 macrophages. (A, B, C) Fold change of mRNA related with glycolysis (A), fatty acid oxidation (B), and TCA cycle (C). qPCR analysis was performed after treatment with DB (9 ng/mL) and/or PMA (2 nM) for 12 h. DB extract promotes the M2-like metabolic reprogramming, decreases glycolysis, and increases FAO and TCA cycle. DB, *Dudleya brittonii* extract; TCA, tricarboxylic acid; PMA, phorbol 12-myristate 13-acetate; qPCR, quantitative polymerase chain reaction. All data represent the mean±standard deviation (n = 3); vs vehicle ^a^ p<0.05, ^b^ p<0.01, ^c^ p<0.001; vs PMA ^d^ p<0.05, ^e^ p<0.01, ^f^ p<0.001.

Along with FAS, FAO plays a key role in the M2-like differentiation of AMs. We therefore performed a qPCR to verify the effect of DB and PMA on the 3 basic energy metabolisms (i.e., glycolysis, FAO, and TCA cycle) in 3D4/31 macrophages. For this assay, we measured the 27 gene expression levels in 3D4/31 macrophages after exposure to DB and/or PMA for 12 h. Treatment with DB alone resulted in decreased expression of glycolysis genes (glucose transporter 1 [*GLUT1*], hexokinase [*HK*] 2, *HK3*, phosphofructokinase platelet [*PFKP*], phosphofructokinase liver [*PFKL*], pyruvate kinase liver and red blood cells [*PKLR*], pyruvate kinase muscle [*PKM*]), and increased expression of FAO genes (long- chain acyl-CoA dehydrogenase [*ACADL*], acetyl-CoA acyltransferase 1A [*ACAA1*], acetyl-CoA acyltransferase 2 [*ACAA2*], peroxisome proliferator-activated receptor α [*PPARA*]) and TCA cycle genes (dihydrolipoamide S-acetyltransferase [*DLAT*], citrate synthase [*CTS*], isocitrate dehydrogenase 3 alpha [*IDH3*], succinate thiokinase [*SUCLG1*], malate dehydrogenase [*MDH*] 1, *MDH2*), as compared to the vehicle (Figure 4A, 4B, and 4C, respectively; p<0.05). This result suggests that DB induces an M2-like metabolic change in 3D4/31 macrophages that are not yet activated. However, the expression levels of glycolysis, FAO, and TCA cycle genes were not significantly changed when 3D4/31 macrophages were activated by PMA treatment for 12 h; only a small number of gene expressions (*ACACA*, *CTS*) were increased, as compared to the vehicle. Finally, co-treatment with PMA and DB resulted in enhanced reduction in the expression of glycolysis genes (*GLUT1*, *HK2*, *HK3*, *PFKP*, *PFKL*, *PKLR*, *PKM*), and increase in the expression of FAO (carnitine palmitoyltransferase 1a [*CPT1A*], *CPT1B*, *ACADL*, *ACAA1*, *ACAA2*, *PPARA*) and TCA cycle genes (*DLAT*, *CTS*, *IDH3*, *SUCLG1*, *MDH1*, *MDH2*), compared to PMA treatment alone.

In addition, the specific expression of anaerobic glycolysis genes (L-lactate dehydrogenase A-like 6B-like isoform [*LDHAL6B*], lactate dehydrogenase A [*LDHA*]) was confirmed to be increased after DB treatment alone or co-treatment with PMA. Also, an unusual expression of the anaerobic glycolysis genes (*LDHAL6B*, *LDHA*) was observed in the case of DB single-treatment or co-treatment with PMA. M2-like macrophages such as AMs use FAO as an important factor for differentiation [29]; thus, up-regulation of FAO by DB is expected to positively affect the M2-like differentiation without inhibiting activity and function of 3D4/31 macrophages.

## DISCUSSION

**Figure 5:**
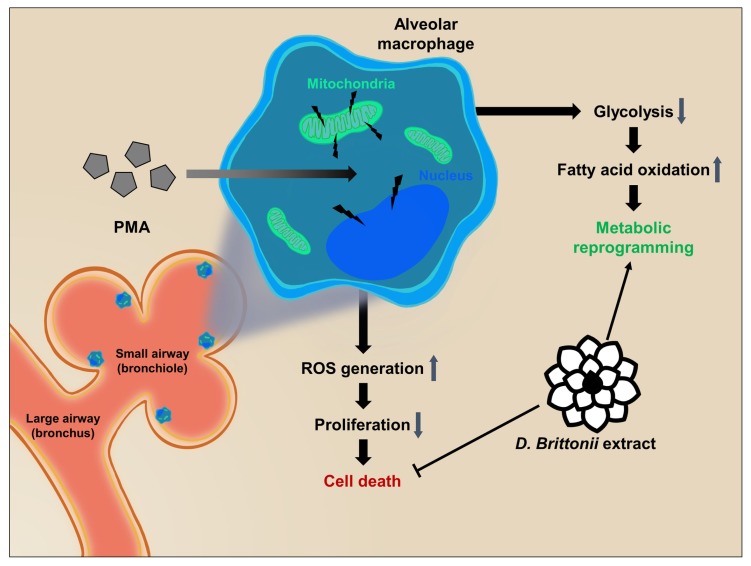
DB upregulates the cell survival rate of 3D4/31 by antioxidant and fatty acid oxidation. Alveolar macrophages are located in bronchioles to protect the lung against infections. DB downregulates the glycolysis gene expression and upregulates the fatty acid oxidation and TCA cycle genes, thereby promoting the M2-like metabolic reprogramming of 3D4/31 macrophages. Moreover, DB reduces macrophage activation (PMA induced) induced ROS generation and cell death *in vitro*. Taken together, DB upregulates the survival rate of 3D4/31 macrophages. DB, *Dudleya brittonii* extract; TCA, tricarboxylic acid; PMA, phorbol 12-myristate 13-acetate; ROS, reactive oxygen species.

In this study, we identified DB to exert an M2-like activation and cell survival rate enhancement effect for AMs *in vitro*. Our results demonstrate that DB inhibits the PMA induced ROS production, cell death, anti-proliferation, and anti-viability effect, and upregulates the FAS, FAO, and TCA cycle pathways in 3D4/31 macrophages (Figure 5). Furthermore, exposure to DB upregulates the specific energy metabolism of FAO and TCA cycle, but not glycolysis. We found further evidence in the role of DB in lipid metabolism during differentiation of porcine AMs. Taken together, our results indicate that DB has the potential to enhance the porcine alveolar immunity to prevent respiratory infectious diseases.

CMs derived from bone marrow possess a 2 to 4 day retention time in the blood [30], and although AMs have a much longer lifetime, their turnover rate is only 40% per year. Therefore, it is very important to keep the existing alveolar macrophage population healthy. Activation of AMs due to respiratory infections is a major threat to the AM population that exists from the time of birth; thus, the survival rate-enhancing efficacy of DB on activated AMs can be helpful for maintaining the respiratory health. AMs function even under normal conditions, even if there is no infection. Most functions of CMs are focused on the phagocytosis of the pathogen, but tissue-specific macrophages play an important role in maintaining homeostasis by removing apoptotic cells or cellular debris. Especially, AMs are closely related to inflammation and various diseases of the respiratory system. Pneumonia, *Pasteurella multocida*, foot-and-mouth disease virus, porcine respiratory coronavirus, porcine reproductive and respiratory syndrome virus, *Streptococcus suis*, *Haemophilus parasuis*, etc., cause great losses in the entire farmhouse because of their high infectivity [31–33]. Therefore, the survival rate enhancement effect of DB on AMs may help to prevent respiratory infectious diseases by improving the respiratory immunity of livestock. In addition, pneumonia is mainly caused by infections such as bacteria and viruses. In pigs, pneumonia easily caused by *Mycoplasma pneumonia*, *Pasteurella multocida*, etc., or complications [34]. Pig pneumonia has a high infection rate and spreads easily on the farms, which inhibit the porcine survival and growth, so proper treatment and prevention are important, but there is no proper treatment or prevention other than antibiotics. However, since the use of feed additive antibiotics is prohibited, a method of improving immunity to prevent pneumonia has been attempted. Since AMs play an important role in the control of pneumonia through anti-inflammatory and phagocytic actions, DB may be useful for the prevention of swine pneumonia [35,36].

Growth hormones and feed additive antibiotics have been used indiscriminately in an age when there is no regulation on livestock production, to promote the growth of livestock and prevent diseases. However, their use has been banned in most countries due to environmental pollution and residue of animal products [37]. As a result, due to the precise correlation between steroid (or antibiotics) and infectious diseases, the repercussion has been an increase in the incidence of diseases in livestock. Hence, the demand for newer methods has increased and is newly developed to replace steroid (or antibiotics) [38]. Currently, steroids can be used for the recovery from bacterial infections or lung diseases, but they have no direct effect on the removal of infections and have been reported to only play a role in helping recovery [39,40]. In this study, we observed increased survival rate of AMs with SRC3 expression, which is a steroid sensor. It has previously been reported that steroid treatment prevents livestock diseases, and lung infections are improved by steroid sensor stimulation such as SRC3 of AMs.

This study confirms that DB upregulates the FAS, FAO, and TCA cycle genes, as well as SRC3 mRNA expression and cell survival rate in 3D4/31 AMs. However, the reason for the increase in LDHA and the decrease in WST-1 cell viability due to DB treatment is difficult to explain. These irregular results are expected to be induced by anaerobic glycolysis [41] or antioxidant effect. WST-1 assay is based on the activity of mitochondrial dehydrogenase (M-DHase) [42], M-DHase carries out energy production through oxidative phosphorylation on mitochondria, which is the largest part of intracellular ROS production [43]. Therefore, if the antioxidant activity of DB is accompanied by the inhibition of M-DHase activity, it is expected that similar results can be obtained as a decrease of viability with the decrease of ROS. In addition, further research in respiratory immunity using small animals such as a mouse needs to be performed *in vivo*, before practical application to livestock. Despite these limitations, this study confirms a new mechanism for the association of lipid metabolism and survival enhancement in the porcine AMs, and suggests that administering DB could potentially improve the survival rate of AMs. This study provides new insights and directions for further research in the immunity of the porcine respiratory system based on AMs and natural materials.

## Figures and Tables

**Table 1:** Primer sequences for fatty acid synthesis, fatty acid oxidation, glycolysis, and tricarboxylic acid cycle

Target genes	Sequences (5′ to 3′)
*FASN*	F: GTGGAGGTGCGCCAGATACT
(Fatty acid synthase)	R: CCTCGTGGGATGTGGGAGTC
*ACACA*	F: ACAGCTGACGGAGGAAGACG
(Acetyl-CoA carboxylase alpha)	R: GCTCGCTGAGTGGGTGAGAT
*SRC3*	F: TCAAAGGCCAGCCGAATGGA
(Steroid receptor coactivator-3)	R: CCAGGCCGCATTTGAAGCAT
*SRA1*	F: AGCTTCCAGGCCTCCACTTG
(Steroid receptor RNA activator 1)	R: GCCAGGCGTCGGTTTATGTC
*GLUT1*	F: CTGCTCATCAACCGCAATGA
(Glucose transporter 1)	R: GGCTCTCCTCCTTCATCTCC
*HK2*	F: CACTGCTGAAGGAAGCCATC
(Hexokinase2)	R: GGGTCTTCATAGCCACAGGT
*HK3*	F: CTCTGGAGGTGTGCAGATCA
(Hexokinase3)	R: TTCTGCTGGAAGTCCACGAT
*PFKP*	F: CCGACGGACACAAGATGTTC
(Phosphofructokinase platelet)	R: TTGTCCCAAGAATGGAGCCT
*PFKL*	F: GAAACGAGAAGTGCCACGAA
(Phosphofructokinase liver)	R: TACCGTAGTTCCGGTCGAAG
*PKLR*	F: AGACTGCCAAGGGTCACTTT
(Pyruvate kinase liver & red blood cells)	R: CAGCTCCTCAAAGAGTTGCC
*PKM*	F: GATCCCAAGACTCTGGCCTT
(Pyruvate kinase muscle)	R: CCAGCTTGTCCATCTGCTTC
*LDHAL6B*	F: GATTGGGCAGAGGCTTGGTA
(L-lactate dehydrogenase A-like 6B-like isoform)	R: CACCAGCGATGTTCACTCCA
*LDHA*	F: TAATGGGGGAAAGGCTGGGA
(Lactate dehydrogenase A)	R: CGCTCCATACAGGCACACTA
*CPT1A*	F: CAAGATAGCGGCCGAAAAGC
(Carnitine palmitoyltransferase 1a)	R: GATAATCGCCACGGCTCAGA
*CPT1B*	F: CCACTATGACCCGGAAGACG
(Carnitine palmitoyltransferase 1b)	R: TTGAACGCGATGAGGGTGAA
*ACADVL*	F: GCGGTGAATCATGCTGCTAA
(Very long-chain acyl-CoA dehydrogenase)	R: GTGGATCCCTGGTCCATGTT
*ACADL*	F: GCGTGGCTTATGACTGTGTG
(Long-chain acyl-CoA dehydrogenase)	R: ACTCGGGCATCCACATAAGC
*ACAA1*	F: TCGCCCAGTTTCTGAGTGAC
(Acetyl-CoA acyltransferase 1A)	R: CCACAAGCCATGCCAATGTC
*ACAA2*	F: TCGTGGGCTATTTTGCGTCT
(Acetyl-CoA acyltransferase 2)	R: TCCTGCTTTCTTCAGTGCCC
*PPARA*	F: TTGAACGACCAGGTCACGCT
(Peroxisome proliferator-activated receptor α)	R: GGAACTCGCGCGTGATGAAG
*DLAT*	F: AGCTTCAGCCTTGGCATGTT
(Dihydrolipoamide S-acetyltransferase)	R: TGCTGACTGCGACACTGATA
*CTS*	F: GGGCACTGGGTGTATTAGCA
(Citrate synthase)	R: TCATGGACTTGGGCCTCTCT
*IDH3*	F: CGCTGCAAAGATTGAGACCG
(Isocitrate dehydrogenase 3 alpha)	R: TCTGAGCATTTTGCGTTGCC
*SUCLG1*	F: CAGGGCACCTTTCATAGCCA
(Succinate thiokinase)	R: CCTTTCCCTGGAGTGGTTCC
*MDH1*	F: TGGTGTTCCTGATGATCTGCTC
(Malate dehydrogenase1)	R: CCTTTGCAGTGAGGTCCATCTT
*MDH2*	F: CAACCCGGTTAACTCCACCA
(Malate dehydrogenase2)	R: ACCCTTCAGCTCTGCAACAA
*GAPDH*	F: GTCGGAGTGAACGGATTTGGC
(Glyceraldehyde 3-phosphate dehydrogenase)	R: ACTGTGCCGTGGAATTTGCC

F: forward; R: reverse.
